# Risk factors of gallbladder cancer in Nepal: A case control study

**DOI:** 10.1371/journal.pone.0317249

**Published:** 2025-01-22

**Authors:** Chanda Thakur, Roshan Kumar Mahato, Sabina Marasini, Dinesh Timalsena, Krishna Sagar Sharma, Biraj Man Karmacharya

**Affiliations:** 1 Department of Public Health, Kathmandu University Dhulikhel Hospital, Dhulikhel, Nepal; 2 Faculty of Public Health, Khon Kaen University, Khon Kaen, Thailand; 3 Department of Medical Oncology, Bharatpur, Chitwan, Nepal; Mayo Clinic in Arizona, UNITED STATES OF AMERICA

## Abstract

**Background:**

Gallbladder cancer (GBC) is a rare, highly fatal disease with diagnosis in advanced stage and low survival rate. Nepal ranked 4^th^ position with highest rates of GBC for 10 countries in 2020.

**Objective:**

To find the association between socio-demographic, behavioral and environmental factors associated with the development of GBC.

**Method:**

A case-control study was conducted in 2021/22 with newly diagnosed gallbladder cancer cases from three cancer-specialized hospitals and one tertiary (superspeciality) hospital. Controls were selected from the same tertiary hospital and one additional hospital providing services to gallbladder pathologies for a huge population, making a total of five hospitals involved in the study. The ratio of cases to control was 1:1. The data collection was done through telephonic interviews using structured questionnaires. The risk factors for GBC were assessed by using unconditional logistic regression to find odds ratios and 95% confidence level for bivariate and multivariate analysis. The statistical analysis was carried out in STATA 18.

**Result:**

A total of 240 respondents were enrolled in the study, among them half were GBC patients (cases) and half were gallbladder patients (controls). The average age of the respondents was 54.82±12.3 years, with female preponderance among both groups. On multivariate analysis, the risk factors studied were; parity ≥3 (AOR = 2.80, 95% CI: 1.17–6.66, P value 0.020), being ethnic group of Terai/Madhesi (AOR = 7.88, 95% CI: 3.16–19.66, P value <0.001), being Janajati (AOR = 3.36, 95% CI: 1.17–6.61, P value <0.001), having gallbladder related disease (AOR = 2.00, 95% CI:1.00–4.02, P value 0.049), consuming alcohol ≥100ml/day (AOR = 3.44, 95% CI:1.11–10.63, P value 0.032), exposed with pesticides ≥2 times in a year (AOR = 4.04, 95% CI: 1.27–12.89, P value 0.018) and consuming less vegetables and fruits (<1 times per day in a week) (AOR = 2.69, 95% CI:1.34–5.40, P value 0.005).

**Conclusion:**

The study reveals key GBC risk factors, offering vital insights for targeted screening, resource allocation, and public health measures to mitigate risks in Nepal.

## Introduction

Gallbladder cancer, the third most common cancer in the bile duct, is very serious with less than 10% of people surviving for five years after diagnosis [1]. Globally, gallbladder cancer is the 25^th^ most common type of cancer [2]. In 2020, there were 115,949 new cases, leading to 84,695 deaths. The incidence and mortality rates of gallbladder cancer vary widely worldwide, with a heavier burden observed in developed countries and among females [3]. Continents with moderate prevalence of gallbladder cancer include South America, East Asia, and Central Europe. Additionally, countries in the Indian subcontinent such as Pakistan, Nepal, Bangladesh, and Bhutan have reported high incidences of gallbladder cancer [4].

GBC is more common in Nepal, more among females and younger patients often presenting with pain in the abdomen and jaundice [5,6]. The Population Based Cancer registry (PBCR, 2019) revealed GBC ranked in fifth position among all sites cancer in Kathmandu Valley with ASR among male and female of 3.7 and 6.7 per 100,000 respectively [7].

Identified risk factors for gallbladder cancer include socio-demographic factors, gallbladder pathologies/abnormalities, exposure to chemicals and smoke, and infections [8,9]. Moreover, these risk factors are further classified as either modifiable or non-modifiable [10]. Dietary patterns influence gallbladder cancer risk, with protective effects linked to the consumption of vegetables and fruits, while the intake of red meat, red chilies, and fried foods with mustard oil contributes to its development [11,12]. Nepalese farmers, in an agricultural setting, face health risks from pesticide exposure, causing acute toxicity, chronic diseases, and neuropathic conditions. Studies, including interventions in Nepal, emphasize the need to explore the link between pesticide exposure and gallbladder cancer [13].

Despite a rising number of GBC cases, few studies have been conducted to explore the risk factors of gallbladder in Nepal [14]. Currently, everyone has an equal chance of developing gallbladder cancer due to unpredictable/unknown risk factors. Disease incidence alone reveals the number of affected individuals but need of emphasis is on accurate information on both incidence and risk factors for effective prevention [4].

Identifying gallbladder cancer risk factors promptly can save lives. This study aims to fill a critical research gap in the country by focusing on hospital patients to investigate and better understand the risk factors associated with GBC. The findings from this research could serve as a valuable reference for future studies. If the associations identified are deemed significant, they could help inform the development of targeted programs addressing GBC risk factors, providing a basis for further research to contribute for prevention and early detection efforts in the future.

## Methods

### Study design and setting

**Fig 1:**
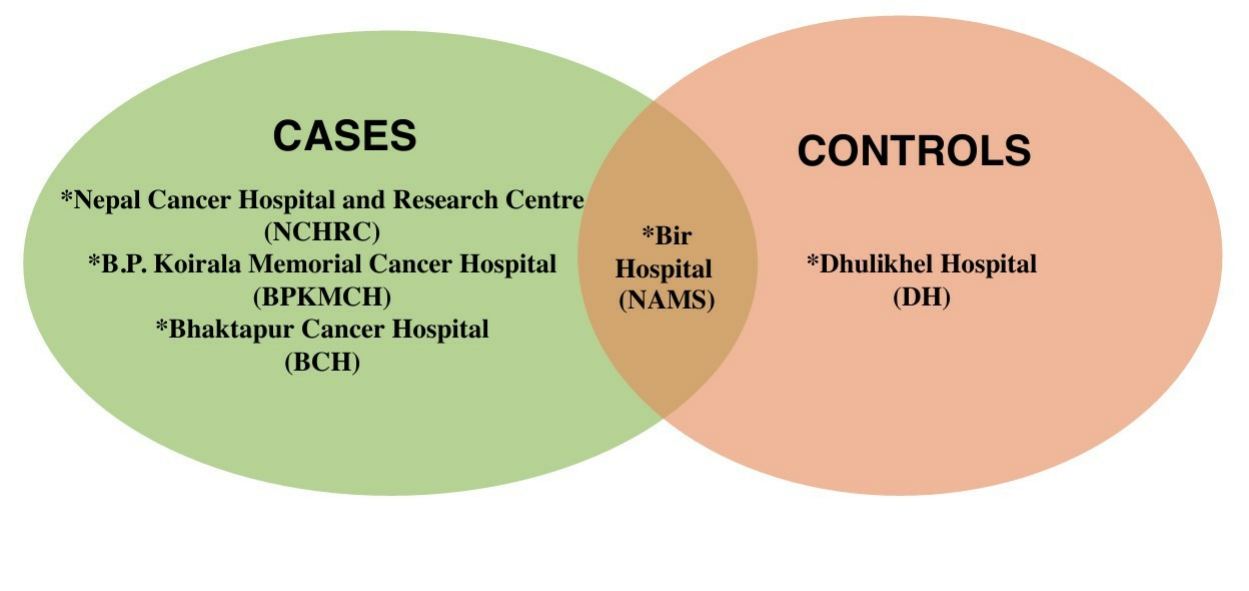
Selection of hospitals for cases and controls.

We conducted a hospital based unmatched case control study. The cases were the Gallbladder cancer patients and controls were benign or nonmalignant/non-cancerous individuals of gall bladder pathologies identified based on the histopathologic reports and diagnosis of the year 2021/22. The research was conducted in 5 Hospitals of Nepal i.e., National Academy for Medical Sciences (NAMS), which is also popular as Bir hospital, Nepal cancer Hospital and research Centre (NCHRC), Bhaktapur cancer Hospital (BCH), B.P Koirala Memorial Cancer Hospital (BPKMCH) Bharatpur and Dhulikhel Hospital. These hospitals were the specialized hospitals in which patients from all around the country visit to have the cancer diagnosis and treatment. Hence, we have traced the patients from all over the country for the expected risk factors. These specialized centers attract diverse patients from across the country, minimizing location-based differences in patient characteristics. Dhulikhel Hospital is recognized for its expertise in gallbladder care, while Bir Hospital served as a common site for both cases and controls, enhancing the study’s generalizability. The selected hospitals for cases and controls are illustrated in the Venn diagram in **Fig 1**.

To reduce potential selection bias from the differing settings, we selected cases and controls from various specialized hospitals across Nepal, ensuring that our sample represents a wide geographical and demographic range. Cancer-specialized hospitals, including Nepal Cancer Hospital and Research Centre, Bhaktapur Cancer Hospital, and B.P. Koirala Memorial Cancer Hospital, were used to recruit cases, while Dhulikhel Hospital and Bir Hospital (both recognized for gallbladder care) were used for controls to ensure access to a population with benign gallbladder conditions. While this approach may introduce setting differences, it was necessary to enhance the representativeness of the case and control groups and ensure a broad range of exposures relevant to the Nepalese population.

### Sample size

The sample size of 240 was calculated by applying the formula of case control study for sample size estimation with the help of OpenEpi software (sample size calculation for unmatched case control study), version (3.03.17) [15]. Among the total sample half i.e. 120 were cases and half i.e.120 were control. The desired confidence level was 95% with 80% power, ratio of controls to cases were 1:1, and the odds ratio from the previous similar study was observed 2.22 which was taken with reference of exposure to vegetable (capsicum). Also, the proportion of case with exposure was 0.531 and proportion of control with exposure was 0.277 [12].

### Sampling technique

Firstly, 4 hospitals offering cancer diagnosis as well as treatment were selected. Among the 4 hospitals, 3 were cancer specialized hospital as NCHRC, BCH, BPKMCH Hospital and 1 tertiary hospital (Bir hospital) which is oldest superspeciality hospital and recently started oncology services as well. From each selected hospital the total number of cases was identified for the year 2021 and 2022 A.D from various departments. All the patients in the list were contacted taking the consent. Among all contacted, many of them were found dead and some were not willing to participate. A total of 509 cases were contacted from the list of contact number received from the hospitals. Among the 509 participants, the total of 120 samples was included in the study by the systematic random sampling method.

During the process of systematic random sampling, we encountered several challenges. Although the initial sample size was based on 509 cases, some patients were unreachable due to invalid or disconnected contact numbers. Additionally, some patients had unfortunately passed away, and a few did not respond despite multiple attempts (3–5 contact attempts) over a week, a follow-up was conducted after 15 days to reattempt contact.

**Fig 2:**
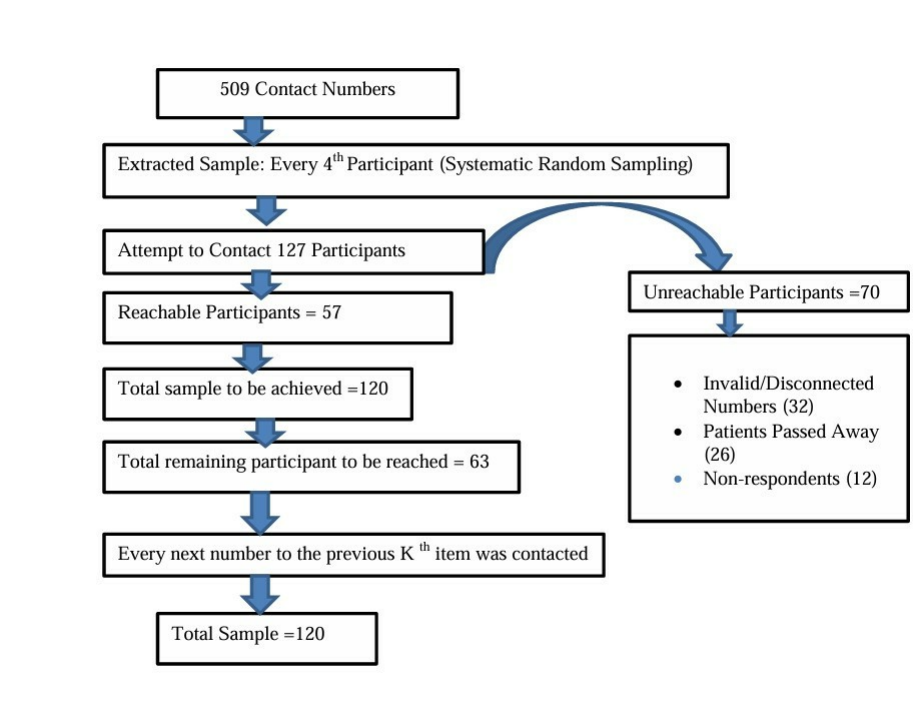
Cases enrolment flowchart.

To address these issues, we followed the systematic random sampling approach, but when a selected case could not be reached or had passed away, we replaced that case with the next eligible participant according to the sampling interval. This ensured that the final sample size of 120 participants was achieved while maintaining the randomness and representativeness of the sample. The process for the enrolment of cases is illustrated in the diagram in **Fig 2**.

Similar process was adopted for selecting controls from the selected hospital. The two hospitals from where controls were selected were Dhulikhel Hospital, Kavre and National Academy for Medical Sciences (NAMS, Bir Hospital) Kathmandu.

Controls were traced from the histopathology report of the patient from the pathology department of the specified hospitals. Those nonmalignant or benign diagnosed gallbladder pathologies patients were further traced for contact details for conducting interview.

### Populations/Participants selection criteria

#### Selection of case

Patients diagnosed with gallbladder cancer in 2021 and 2022 who were aged 18 years or older were eligible for inclusion. Patients were excluded if they were under 18 years of age, had a diagnosis other than gallbladder cancer, or were unable/unwilling to participate due to physical or mental incapacity.

#### Selection of control

Control participants were patients diagnosed in 2021 and 2022 with benign gallbladder-related pathology (i.e., not gallbladder cancer) and aged 18 years or older. Exclusion criteria were same as those of the case group, with additional exclusion for patients whose histopathologic diagnosis was unrelated to the gallbladder site.

### Data collection tools

The data collection was started from 15th July to 31st October, 2022.The study tool was divided into five sections: a) sociodemographic characteristics; b) smoking and alcohol consumption; c) physical activity; d) pesticide exposure; e) food pattern. The tool was confirmed and approved after frequent literature reviews and multiple consultations with the experts.

#### Assessment of socio-demographic characteristics

The standard questionnaire of the Non Communicable Disease (NCD) risk factor step survey [16] was used for demographic details, with some modifications after pretesting. The questionnaire includes information about the demographic characteristics, with a major focus on age, sex, marital status, ethnicity, occupation, number of children, annual family income, place of residence, as well as disease related to the gallbladder, history of exposure to disease, chemicals, rubber, and textiles.

#### Assessment of smoking and alcohol consumption

Smoking-related questions were adopted from the Global Adult Tobacco Survey (GATS) Core Questionnaire with Optional Questions and the NCD Step Survey [16,17]. The GATS Core Questionnaire primarily focuses on tobacco use, including current smoking status, frequency, and the types of tobacco products used. However, only smoking-related questions have been included in the questionnaire. Based on this, participants were categorized as nonsmokers, current smokers, and former smokers. In the GATS questionnaire, there were eight sections aimed at collecting information on background, knowledge, attitudes, and practices related to smoking and alcohol. However, only the sections addressing smoking-related questions were included. Additionally, from Section C, questions about smokeless tobacco were asked, with modifications to account for local names of cigarette types in Nepal. Alcohol-related questions were taken from the NCDs step survey, and the cut-off value for alcohol consumption was taken from the WHO report: <100 mL and >100 mL [16,18].

#### Assessment of physical activity

The standard tool of Guidelines for Data Processing and Analysis of the International Physical Activity Questionnaire (IPAQ) was used to calculate physical activity. Taking the reference from IPAQ analysis, the cut-off values were categorized into <3000 MET minute/week as low active and >3000 MET minute/week as highly active [19].

#### Assessment of pesticides exposure

For pesticide measurement, pesticide exposure intensity as well as cumulative pesticide exposures were calculated by the standard formula [20]. Information about practices and pesticide use was gathered by asking questions about exposure to pesticides, frequency of exposure within a year, frequency of mixing and applying pesticides, types of pesticides used, and the extent of involvement in repairing spraying equipment. Additionally, respondents were asked whether they used protective measures. Based on this information, the intensity level of pesticide exposure was calculated using a standard formula:

Intensity level = (mix+ Application +Repair) *PPE

‘Mix’ explains the mixing status; ‘Application’ explains the application method applied to various instruments; ‘Repair’ implies whether the participants got involved in the repair of the instrument used or not; and ‘PPE’ indicates the various forms of PPE that have been applied during the pesticide’s sprays. The detailed categories of these terms are included in the questionnaire form.

In the next step, the intensity level was used to calculate the cumulative exposure of the pesticides and is given by the formula:

Cumulative Exposure = Intensity level * Duration*frequency

In the above equation, the intensity level is calculated by the above formula; duration is the time period, which was taken as 1 year before the diagnosis of disease; and frequency is the number of times the participants are exposed to the pesticides. The value of the cumulative exposure result was in the score, which was in continuous data, so the median value was taken as the cutoff point value and categorized as less than and greater than 2 times exposure with pesticides 1 year before the diagnosis of disease.

#### Assessment of food pattern

The validated tool of the Food Frequency Questionnaire (FFQ) of the Dhulikhel Heart Study (DHS) was used to assess the dietary intake [21]. All responses were asked about the period one year before the diagnosis of the disease.

### Data collection techniques

Gallbladder cancer, being a rare disease, often requires patients in advanced stages to stay at home, visiting hospitals only for emergencies, follow-up treatments, or surgeries. Consequently, we conducted telephonic interviews, adapting our approach to each patient’s condition. We initially contacted participants via their personal cell numbers; in some cases, patients answered directly, while in others, relatives received the call. We first explained our research objectives emphasizing the long-term importance of gathering this information for the population and clarified that it does not provide immediate benefits. Verbal consent was obtained and recorded before beginning the interview. Also, Interviews, which typically lasted between 30 to 50 minutes, were scheduled at times when the patients were most comfortable. To ensure their comfort, the interviews were divided into shorter segments, such as splitting a 50-minute interview into two 25-minute sessions, helping to reduce fatigue and maintain participant ease throughout the process. When patients were too weak to respond, we gathered information from their relatives or caregivers, ensuring that the patients were present during the data collection process.

### Validity and reliability

Pre-testing of questionnaires was done by taking the 12 cases from Bhaktapur Cancer Hospital, and the 12 controls were selected from Dhulikhel Hospital. The collected data were checked and verified on the same day of data collection. Following the pretesting, few modifications were made in the study tools to ensure consistency and maintain the flow of questions.

### Data analysis

Data were entered in the Kobo Toolbox [22], and the entered data were transferred to Stata (version 18) for analysis. The categorical data were reported as frequency and percentage. The mean, standard deviation, median, and range (minimum and maximum) were described for the continuous variable. After the descriptive analysis of the general characteristics of the cases and controls, odds ratios (OR) and their 95% confidence intervals (CI) were estimated using unconditional logistic regression for the binary outcome of being classified as either a case (diagnosed with GBC) or a control (not diagnosed with GBC).Bivariate analysis was performed to measure the effect of each variable of interest on risk factors for sociodemographic variables, pesticide exposure, and food patterns. Multivariate analysis was performed by multiple logistic regression including variables that showed a significant statistical association for the risk factors of GBC in bivariate analysis. Variables associated in the bivariate analysis with P value (≤ 0.25) were included in the final model.

### Ethical approval

Ethical clearance was obtained from the Institutional Review Committee, Kathmandu University School of Medical Sciences (Reference Number: IRC62/22, approval date: June 19, 2022), and ethical clearance from the Ethical Review Board of the Nepal Health Research Council (NHRC) was also obtained (Reference Number: 4528, approval date: July 13, 2022). Study permission was also obtained from assigned hospitals before conducting the study. Due to the telephonic interview, it was not possible to get written consent from each respondent; therefore, verbal consent was obtained from the respondents during data collection. The verbal/oral consent was recorded during the interview, using mobile recorder during the conversations in the telephonic interview.

## Results

### Characteristics of gallbladder cancer patients (Case) and gallbladder disease patients (Control)

The mean age of participants was 53.9 ± 12.1 years. Female participants accounted for over 60% of both groups, with 67.5% in cases and 72.5% in controls. More than 40% of the participants had no formal education, with 60.8% in the case group and 41.6% in the control group. Agriculture was the primary occupation for participants, with 42.5% of cases and 35.0% of controls engaged in this field.

The annual family income was slightly higher among controls ($5,091.4 ± 7,476.4) compared to cases ($4,885.5 ± 7,137.8). The majority of participants, both cases (74.2%) and controls (61.6%), reported no gallbladder-related disease one year prior to diagnosis. More than 50% of participants were never smokers, with 55.0% in cases and 65.8% in controls. Additionally, over 85% of participants consumed less than 100 ml of alcohol, with 85.8% in cases and 94.2% in controls.

More than 80% of both groups were exposed to pesticides more than twice in their lifetime, with 95.8% of cases and 83.3% of controls reporting such exposure. Approximately 66.6% of cases were intermediate consumers of vegetables and fruits, while 60.0% of controls were classified as low consumers. The detail characteristics of the cases and controls are shown in Table 1.

**Table 1:** Characteristics of gallbladder cancer patients (Case) and gallbladder disease patients (Control).

Characteristics	Case, n = 120	Control, n = 120
**Age (Years)**		
Mean (SD)	55.6(±12.6)	53.9(±12.1)
**Educational Status, n (%)**		
No formal education	73(60.8)	50(41.6)
Less than higher secondary	17(14.2)	23(19.2)
Higher secondary and more	30(25)	47(39.2)
**Gender, (n %)**		
Male	39(32.5)	33(27.5)
Female	81(67.5)	87(72.5)
**Number of Children among females (Parity),(n %)**		
<3	17(20.9)	36(41.4)
≥6	64(79)	51(58.6)
**Marital Status, (n %)**		
Single (Divorced/Widow)	16(13.3)	7(5.8)
Married	104(86.6)	113(94.2)
**Ethnicity, (n %)**		
Brahmin/ Chhetri	31(25.83)	70(58.3)
Terai/Madhesi(Dalit/Muslim/other)	41(34.2)	8(6.67)
Janajati (Newar)	48(40)	42(35)
**Occupation, (n %)**		
Agriculture	51(42.5)	30(25)
Business/private job/civil services	12(10)	23(19.2)
Unemployed (able to work)/Student	18(15)	45(37.5)
Others	39(32.5)	22(18.33)
**Annual family Income (in USD)**		
Mean (±SD)	4,885.5 (±7,137.8)	5,091.4(±7,476.4)
Median (Min, Max)	3,625.9(38.2, 45,801)	3,816.7(38.2, 49,618)
**Gallbladder Disease (1 year before the diagnosis of disease), (n %)**		
Yes	31(25.8)	46(38.3)
No	89(74.2)	74(61.6)
**Smoking Status, (n %)**		
Former smoker	10(8.3)	11(9.2)
Current Smoker	44(36.6)	30(25)
Never	65(55.0)	79(65.8)
**Alcohol ml/day, (n%)**		
<100ml	103(85.8)	113(94.2)
≥13(94)	17(14.2)	7(5.8)
**Pesticide Exposure (Cumulative Pesticides Exposure), (n %)**		
<2 times/year	5(4.2)	20(16.6)
≥0 times/year	115(95.8)	100(83.3)
**Food Pattern**		
**Vegetable/Fruits (times per day in a week), (n %)**		
Intermediate Consumption (1–2)	80(66.6)	48(40.0)
No/less consumption (<1)	40(33.3)	72(60.0)
**Packaged food (times per day in a week), (n %)**		
No/less consumption (<1)	80(66.6)	56(46.6)
Intermediate Consumption (1–2)	20(16.6)	29(24.2)
High Consumption (≥3)	20(16.6)	35(29.2)

### Factors associated with gallbladder cancer patients (Case) and gallbladder disease patients (Control)

Bivariate analysis revealed a crude association with some of the socio-demographic and behavioral factors. The study showed that those who were married (OR = 2.48, CI: 0.98–6.27, P value 0.045) and had more than three children (OR = 2.65, 95% CI: 1.34–5.26, P value 0.004) had a higher risk of having gallbladder cancer. The study showed a higher risk of developing gallbladder cancer with ethnic variations: Terai/Madhesi (OR = 11.57, 95% CI: 4.86–27.55, P value <0.001) and Janajati (OR = 2.58, 95% CI: 1.42–4.66, P value<0.001). Those who were exposed to gallbladder disease (OR = 1.78, 95% CI: 1.02–3.09, P value 0.037) were more likely to develop gallbladder cancer. Moreover, the study showed those who consumed alcohol (OR = 2.66, 95% CI: 1.06–6.68, P value 0.029) had a two-fold higher risk of developing gallbladder cancer. Furthermore, those who were exposed to pesticides (OR = 4.6, 95% CI: 1.66–12.70, P value 0.001) more than twice in a year were more likely to develop gallbladder cancer. Those who less consume vegetables and fruits (OR = 3, 95% CI: 1.77–6.66, P value < 0.001) were at higher risk of developing gall bladder cancer. The study showed that the participants who had a higher secondary level and above education (OR = 0.43, 95% CI: 0.24–0.78, P value 0.005) were less likely to develop gallbladder cancer. Those who were involved in business (OR = 0.30, 95% CI: 0.13–0.70, P value 0.005) and leisure (OR = 0.23, 95% CI: 0.11–0.47, P value<0.001) were less likely to develop gallbladder cancer. Moreover, the study showed that packaged food was less likely to develop gallbladder cancer. Those who were exposed to intermediate consumption, i.e., 1–2 times per day in a week (OR = 0.48, 95% CI: 0.24–0.93, P value 0.006) and high consumption more than 3 times per day in a week (OR = 0.4, 95% CI: 0.20–0.76, P value 0.006) are shown to be at lower risk compared to those with less consumption (Table 2).

**Table 2:** Factors associated with gallbladder cancer patients (Case) and gallbladder disease patients (Control).

Characteristics	Case, n = 120		Control, n = 120	COR (CI)	P value		AOR (CI)	P value
**Age (Years)**								
Mean (SD)*		55.6 (±12.6)	53.9(±12.1)	1.01(0.99–1.03)	0.277		-	-
**Educational status**								
No formal education		73(60.8)	50(41.6)	Ref				
Less than higher secondary		17(14.2)	23(19.2)	0.50(0.24–1.04)		0.065	-	-
Higher secondary and more		30(25)	47(39.2)	0.43(0.24–0.78)		**0.005** ^**^	-	-
**Gender**								
Male		39(32.5)	33(27.5)	Ref				
Female		81(67.5)	87(72.5)	0.78(0.45–1.37)		0.397	-	-
**Number of children (Parity)**								
<3		17(20.9)	36(41.4)	Ref				
≥e		64(79.0)	51(58.6)	2.65(1.34–5.26)	**0.004** **		2.8(1.17–6.66)	**0.020** **
**Marital status**								
Single(Divorced/Widow)		16(13.3)	7(5.8)	Ref				
Married		104(86.6)	113(94.2)	2.48(0.98–6.27)	**0.045** **		-	-
**Ethnicity**								
Brahmin/ Chhetri		31(25.8)	70(58.3)	Ref				
Terai/ Madhesi		41(34.1)	8(6.6)	11.57(4.86–27.55)	**<0.001** *		7.88(3.16–19.66)	**<0.001** *
Janajati		48(40.0)	42(35.0)	2.58(1.42–4.66)	**0.002** **		3.36 (1.71–6.61)	**<0.001** *
**Occupation**								
Agriculture	51(42.5)		30(25.0)	Ref				
Business	12(10.0)		23(19.2)	0.30(0.13–0.70)	**0.005** **		0.37 (0.14–0.95)	**0.040** **
Unemployed/Student	18(15.0)		45(37.5)	0.23(0.11–0.47)	**<0.001** *		0.19 (0.88–0.44)	**<0.001** *
Others	39(32.5)		22(18.3)	1.04(0.52–2.07)	0.905		1.16 (0.51–2.63)	0.709
**Annual family income**								
<3,800	55(±45.8)		60(±50.0)	Ref				
≥ 3,800	65(±54.2)		60(±50.0)	1.14(0.68–1.89)	0.605		-	-
**Gallbladder disease (1 year before the diagnosis of disease)**								
No	31(25.8)		46(38.3)	Ref				
Yes	89(74.2)		74(61.6)	1.78(1.02–3.09)	**0.037** **		2.00 (1.00–4.02)	**0.049** **
**Smoking status**								
Former smoker	10(8.3)		11(9.2)	Ref				
Current Smoker	44(36.6)		30(25.0)	1.61(0.60–4.27)	0.336		-	-
Never	65(55.0)		79(65.8)	0.91(0.38–2.14)	0.857		-	-
**Alcohol (ml/day)**								
<100	103(85.8)		113(94.1)	Ref				
≥ 100	17(14.2)		7(5.8)	2.66(1.06–6.68)	**0.029** **		3.44 (1.11–10.63)	**0.032** **
**Pesticide Exposure (Cumulative Pesticides Exposure)**								
<2 times/years	5(4.2)		20(16.6)	Ref				
≥e	115(95.8)		100(83.3)	4.6(1.66–12.70)	**0.001** **		4.04 (1.27–12.89)	**0.018** **
**Food Pattern**								
**Vegetable/Fruits (times per day in a week)**								
Intermediate Consumption (1–2)	80(66.6)		48(40.0)	Ref				
Less consumption (<1)	40(33.3)		72(60.0)	3(1.77–5.08)	**<0.001** *		2.69 (1.34–5.40)	**0.005** **
**Packaged food (times per day in a week)**								
Less consumption (<1)	80(66.6)		56(46.6)	Ref				
Intermediate Consumption (1–2)	20(16.6)		29(24.2)	0.48(0.24–0.93)	**0.032** **		-	-
High Consumption (≥3)	20(16.6)		35(29.2)	0.4(0.20–0.76)	**0.005** **		-	-

“Multivariate analysis was only performed for variables with a P value ≤0.25 in the bivariate analysis”.

SD: Standard Deviation; COR: Crude Odds Ratio; CI: Confidence Interval; AOR: Adjusted Odds Ratio.

* Indicates statistical significance (p <0.001).

** Indicates statistical significance (p <0.05).

All variables which had shown possible statistically significant association (P value < 0.25) in the bivariate analysis, were collectively entered in the multivariable analysis, and according to the multivariate logistic regression analysis, seven variables were identified as a risk factors for the development of gallbladder cancer after controlling possible confounders, shown in Table 2.

The risk of developing gallbladder cancer was 2.8 times higher among those who had more than three children (AOR = 2.80, 95% CI: 1.17–6.66, P value 0.020) compared to those who had fewer than three children, adjusting for occupation, ethnicity, gallbladder-related disease, cumulative pesticide exposure, and fruit and vegetable consumption. Similarly, the risk of development of gallbladder cancer was 7.8 times higher among the Terai/Madhesi (AOR = 7.88, 95% CI: 3.16–19.66, P value <0.001) and 3.36 times higher among the Janajati (AOR = 3.36, 95% CI: 1.17–6.61, P value <0.001) compared to Brahmin/Chhetri. Compared to those who didn’t have gallbladder-related disease in the past, the risk of developing gallbladder cancer was two times higher among those who had gallbladder-related disease in the past (AOR = 2.00, 95% CI: 1.00–4.02, P value 0.049). Compared to those who were exposed less than 2 times to the pesticides during their lifetime, the risk of developing gallbladder cancer was 4 times higher among those who were exposed greater than 2 times to pesticides during their lifetime (AOR = 4.04, 95% CI: 1.27–12.89, P value 0.018). Compared to those who do not consume alcohol, alcohol consumers had a 3.44 times higher risk of developing gallbladder cancer (AOR = 3.44, 95% CI: 1.11–10.63, P value 0.032). Compared to intermediate consumer of vegetable and fruits the risk of development of gallbladder cancer was 2.69 times higher among less consumer of vegetables and fruits compared to intermediate consumer (AOR = 2.69, 95% CI:1.34–5.40, P value 0.005). In relation to those were engaged in agriculture, the risk of development of gallbladder cancer was 0.19 times lower among unemployed though able to work (AOR = 0.19, 95% CI: 0.88–0.44, P value < 0.001).

## Discussion

In our study on gallbladder cancer risk factors, a significant female preponderance was observed, with a male-to-female ratio of approximately 1:2, consistent with findings from a previous study from Nepal reporting a higher GBC incidence among females [14]. A hospital-based study in Nepal indicated that 72% of GBC patients were female, with a male-to-female ratio of approximately 1:2 [23]. Additionally, a study at Nepal Medical College, Kohalpur, and a cancer registry study reported an even higher female preponderance, with a male-to-female ratio of 1:3 [6,24]. Our analysis found, having three or more children as an associated risk factor for GBC. This finding is consistent with similar study done for GBC suggesting that an elevated risk of GBC in females may be linked to reproductive factors. Hormonal influences and reproductive history, such as early or late age at menarche, are thought to contribute to this increased risk. Additionally, higher parity could lead to bile stasis during pregnancy, which may exert a toxic effect on the gallbladder mucosa, potentially promoting GBC development [25].

Our analysis identified significant ethnic variation in GBC risk, with Terai/Madhesi and Janajati groups exhibiting higher risk of development of GBC compared to the Brahmin/Chhetri group. Specifically, the Terai/Madhesi group showed markedly higher odds of GBC, with an adjusted odds ratio of 7.88, while Janajati individuals had an adjusted odds ratio of 3.36, both of which were statistically significant. These ethnic differences in GBC risk align with findings from the National Cancer Database, sponsored by the American Cancer Society, which also reported notable variations in GBC incidence across different ethnic groups [26]. Similar results for variations in ethnicity were shown worldwide, as certain ethnic groups like Hispanics, American Indians, Mexican Indians, Alaskan natives, and Asian Indians were found to have a higher than normal risk for the development of GBC [27].One of the research suggests that these variations may be influenced by both environmental and geographical factors, including exposure to natural resources like river belts and the quality of water in these areas [28]. This result was consistent with the systematic review article (Gallbladder Cancer in the 21st Century), which observed striking geographical variability in the prevalence of GBC worldwide. These factors are considered to be attributed to differences in environmental exposures and a regional intrinsic predisposition to carcinogenesis in that review [29].

Occupation is found to be associated with the GBC, and those who are unemployed and engaged in private jobs, as well as some other occupations, are shown to have a lower risk of developing the GBC. No particular study related to occupation or GBC has been found yet.

Overall, our study identifies several socio-demographic factors associated with the development of gallbladder cancer, including reproductive history, ethnicity, and occupation. These factors provide a comprehensive understanding of the risk profile for GBC in the studied population.

The behavioral characteristics of the patients were found to be associated with the development of GBC in this study. Specifically, alcohol consumption was identified as a factor associated with GBC development. However, a prospective study on the risk of death showed no clear association between alcohol consumption and mortality due to GBC [30].

Regarding environmental or chemical exposure, most cases and controls in our study reported no direct exposure to pesticides, likely due to the hospital-based setting of the study. Among those who were exposed, a significant association was found between cumulative pesticide exposure and the development of GBC. Participants exposed to pesticides two or more times per year had a significantly higher risk of developing GBC. Our findings are consistent with a similar case-control study on organochlorine pesticides in GBC patients. While pesticide levels in blood samples did not differ significantly, the concentration of specific pesticides such as BHC and DDT was notably higher in the bile of GBC patients compared to those with cholelithiasis. This suggests a potential link between pesticide exposure and GBC, as the increased concentration of BHC and DDT in bile may contribute to carcinogenesis in the gallbladder [31].

The 115 food items were categorized into seven groups, and among these, only vegetables and fruits showed a significant association with gallbladder cancer. The analysis indicated that individuals who consume fewer vegetables and fruits have a higher risk of developing GBC compared to those with intermediate consumption levels. This finding is consistent with similar case-control study done in Nepal that suggest vegetable and fruit consumption may help protect against GBC [14]. Additionally, research conducted at the University Hospital, Banaras Hindu University in Varanasi, India, found that a notable reduction in gallbladder cancer risk was associated with the consumption of cruciferous vegetables [12].

Overall, our findings suggest that eating enough vegetables and fruits, may help reduce the risk of gallbladder cancer. More research is needed to understand how diet affects GBC risk. Conducting survival analysis specific to GBC and studying healthcare-seeking patterns and public awareness could further inform prevention strategies, support earlier diagnosis, and improve patient outcomes.

## Strength and limitation of the study

This study found the association of GBC in the context of Nepal by using scientific research as a case control study. Gallbladder cancer being a rare disease, it was difficult to trace the patients, but the study eventually succeeded in finding and conducting interviews with the patients. The present study also generates evidence regarding the associated factors of GBC, which will help increase the population’s knowledge. Furthermore, the study also provides a platform for new studies for further confirmation. Moreover, with rising cases of gallbladder cancer, its risk factors are essential to be identified in a larger context, but since the present study captures samples of the affected population from specialized hospitals, it will help shed some light on the risk factors.

Due to the nature of the study, recall biases are expected to occur, so the finding may depend on the memory capacity of the participants. The probability of having the chance of mixing actions before and after disease occurrence (alcohol, food pattern) is its limitation. Participants in the study could not identify the exact disease diagnosis and responded that they had gallbladder-related problems before. Generalizations are only limited to the patients of the five hospitals included in the study. Selection bias may be present due to differing recruitment settings; cases were from cancer-specialized hospitals, while controls were from hospitals focused on benign gallbladder conditions. This approach ensured controls reflected non-cancer gallbladder issues but may introduce setting-related differences.

## Conclusion

The study identifies both non-modifiable and modifiable risk factors for having gallbladder cancer development. Key findings include significant associations with parity, ethnicity, occupation, previous history of gallbladder disease, alcohol consumption and pesticide exposure which have been associated with gallbladder cancer. These findings can guide future recommendations related to screening, prevention, and early detection strategies. The findings of the study can future recommend for the prioritization of GBC screening for female and other vulnerable groups as well. Prioritization of GBC screening for females and vulnerable groups is required.

## Supporting information

S1 File (XLSX)

S2 File (DOCX)
