# Supplementary material for: Risk factors of gallbladder cancer in Nepal: A case control study
Source: PLoS One. 2025 Jan 22;20(1):e0317249. doi: 10.1371/journal.pone.0317249 (PMC11753635; doi:10.1371/journal.pone.0317249)
Supplement: S2 File — (DOCX) [file pone.0317249.s002.docx]

English Questionnaire

Study Title: Risk Factors of Gallbladder Cancer: A case control study

| Socio-Demographic Factors | | | | |
| --- | --- | --- | --- | --- |
| 1 | | Date |  |  |
| 2 | | Identification Number |  |  |
| 3 | | Consent | 1 | Yes |
|  |  |  | 0 | No |
| 4 | | First Name |  | |
| 5 | | Middle Name |  | |
| 6 | | Title |  | |
| 7 | | age |  | |
| 8 | | Educational Status | 1 | Illiterate |
|  |  |  | 2 | Literate |
|  |  |  | 3 | Primary |
|  |  |  | 4 | Secondary |
|  |  |  | 5 | Higher Secondary |
|  |  |  | 6 | Bachelor level |
|  |  |  | 7 | Masters Level and above |
| 9 | | Sex | 1 | Male |
|  |  |  | 2 | Female |
|  |  |  | 3 | Others |
| 10 | | Number of children? |  |  |
| 11 | | Marital Status | 1 | Unmarried |
|  |  |  | 2 | Married |
|  |  |  | 3 | Divorced |
|  |  |  | 4 | widow |
|  |  |  | 5 | Living together |
| 12 | | Ethnicity | 1 | Brahmin/ Chhetri |
|  |  |  | 2 | Terai/Madhesi |
|  |  |  | 3 | Dalit |
|  |  |  | 4 | Newar |
|  |  |  | 5 | Janajati |
|  |  |  | 6 | Muslim |
|  |  |  | 7 | Others |
| 13 | | Occupation | 1 | Agriculture |
|  |  |  | 2 | Business |
|  |  |  | 3 | Civil Services |
|  |  |  | 4 | Student |
|  |  |  | 5 | Unemployed (able to work) |
|  |  |  | 6 | Unemployed (able to work) |
|  |  |  | 7 | Others |
|  |  |  | 8 | Private job |
| 14 | | Number of Family Members |  | |
| 15 | | Annual Family Income |  | |
| 16 | | Place of residence 1 year before the diagnosis of disease. | District before | |
|  |  |  | Municipality before | |
|  |  |  | Ward before | |
| 17 | | Place of residence at present | District present | |
|  |  |  | Municipality present | |
|  |  |  | Ward present | |
| 18 | | Do you have disease related to Gallbladder 1 year before the diagnosis of disease? | 1 | Yes |
|  |  |  | 0 | No |
| 19 | | Do you have problem of gallstone 1 year before the diagnosis of disease? | 1 | Yes |
|  |  |  | 0 | No |
| 20 | | Do you have problem of Cyst 1 year before the diagnosis of disease? | 1 | Yes |
|  |  |  | 0 | No |
| 21 | | Do you have history of typhoid 1 year before the diagnosis of disease? | 1 | Yes |
|  |  |  | 0 | No |
| 22 | | Were you been exposed to rubber and textiles industries 1 year before the diagnosis of disease? | 1 | Yes |
|  |  |  | 0 | No |
| 23 | | Do you have history of Cancer in Family | 1 | Yes |
|  |  |  | 0 | No |
| 24 | | History among Family members | 1 | Grandfather/Grandmother |
|  |  |  | 2 | Mother/Father |
|  |  |  | 3 | Brother/Sister |
|  |  |  | 4 | Father's brother/ sister |
|  |  |  | 5 | Mother's brother/ sister |
|  |  |  | 6 | others |
| 25 | | Smoking Related Questions | | |
| i | | Do you smoke at Present? | 1 | Daily |
|  |  |  | 2 | Less than daily |
|  |  |  | 0 | Not at all |
|  |  |  | 4 | Don't Know |
|  |  |  | 5 | Refused |
| ii | | Did you smoke daily 1 year before the diagnosis of disease? | 0 | No |
|  |  |  | 2 | Don't Know |
|  |  |  | 3 | Refused |
|  |  |  | 4 | Never |
| iii | | How old were you when you first started smoking? |  | |
|  |  |  |  |  |
|  | | Age |  | |
| iv | | How many years ago did you first started smoking? |  | |
| Smoking Type | | | | |
| a | Manufactured cigarettes | | | |
|  | Per day | |  |  |
|  | Per week | |  |  |
|  | Cigar | | | |
| b | Per day | |  |  |
|  | Per week | |  |  |
|  | Bidi | | | |
| c | Per day | |  |  |
|  | Per week | |  |  |
|  | Pipe | | | |
| d | Per day | |  |  |
|  | Per week | |  |  |
|  | | | | |
| e | | Per day |  |  |
|  | | Per week |  |  |
|  | | Hookah/Sisah | | |
| f | | Per day |  |  |
|  |  | Per week |  |  |
| v | | Time of taking first smoke after the wake up 1 year before the diagnosis of disease | 1 | Within 5 minutes |
|  |  |  | 2 | Within 6 to 30 minutes |
|  |  |  | 3 | 31 to 60 minutes |
|  |  |  | 4 | More than 60 minutes |
|  |  |  | 5 | Refused |
| vi | | How long it has been since you quitted smoking? | Year |  |
|  |  |  | Month |  |
|  |  |  | Days |  |
|  |  |  | Week |  |
|  |  |  | Less than 1 day |  |
| 26 | | Alcohol | | |
| a | | Had you been drinking alcohol at (present) this month? | 1 | Yes |
|  |  |  | 0 | No |
| b | | Had you been drinking alcohol 1 year before the diagnosis of disease? | 1 | Yes |
|  |  |  | 0 | No |
| c | | Did you drink jaad 1year before you were diagnosed? | 1 | Yes |
|  |  |  | 0 | No |
|  | | Did you drink wine before you were diagnosed? | 1 | Yes |
|  |  |  | 0 | No |
| d | | Did you drink alcohol/hard drinks before diagnosis? | 1 | Yes |
|  |  |  | 0 | No |
| e | | How much jaad did you drink before you were diagnosed? |  |  |
| f | | How much wine did you drink before you got sick? |  |  |
| g | | How much alcohol/hard drinks did you drink before you were diagnosed? |  |  |
| 27 | | Physical activity | | |
| Vigorous type of during those days 1 year before the diagnosis of disease?** | | | | |
| a | | were you doing vigorous type of physical activity 1 year before the diagnosis of disease. | 1 | Yes |
|  |  |  | 0 | No |
| b | | How many days a week did you do vigorous work 1 year before the diagnosis of disease? | Days per week |  |
|  |  |  | Not Done |  |
| c | | vigorous type of during those days 1 year before the diagnosis of disease?** | hours per day |  |
|  |  |  | minute per day |  |
|  |  |  | Don't Know |  |
| Moderate type of work during those days 1 year before the diagnosis of disease? | | | | |
| d | | were you doing moderate type of physical activity 1 year before the diagnosis of disease. | 1 | Yes |
|  |  |  | 0 | No |
| e | | How many days a week did you do moderate work 1 year before the diagnosis of disease? | Days per week |  |
|  |  |  | Not Done |  |
| f | | Moderate type of work during those days 1 year before the diagnosis of disease? | hours per day |  |
|  |  |  | minute per day |  |
|  |  |  | Don't Know |  |
|  | | Walking | | |
| g | | Were you spending time on walking 1 year before the diagnosis of disease? | 1 | Yes |
|  |  |  | 0 | No |
| h | | How many days do you spend on walking at least for 10 minutes in a week 1 year before the diagnosis of disease?** | hours per day |  |
|  |  |  | minute per day |  |
|  |  |  | Don't Know |  |
| sitting | | | | |
| i | | were you spend time by sitting 1 year before the diagnosis of disease? | 1 | Yes |
|  |  |  | 0 | No |
| j | | How much time did you spend sitting on a week day 1 year before the diagnosis of disease?** | hours per day |  |
|  |  |  | minute per day |  |
|  |  |  | Don't Know |  |
| 28 | | Pesticides Exposure | | |
| a | | Have you ever been exposed to pesticides? (1 year before the diagnosis of disease) | 1 | Yes |
|  |  |  | 0 | No |
| b | | If yes, How many times in a year (1 year before) | Day |  |
|  |  |  | week |  |
|  |  |  | Month |  |
| c | | Have you ever mixed the pesticides? (1 year before the diagnosis of disease) | 1 | Yes |
|  |  |  | 0 | No |
| d | | Have you ever used instrument to apply pesticides 1 year before the diagnosis of disease? | 1 | Yes |
|  |  |  | 0 | No |
| e | | Types of instruments used during spray 1 year before the diagnosis of study | 1 | Aerial aircraft |
|  |  |  | 2 | seed treatment |
|  |  |  | 3 | Distribute tablets |
|  |  |  | 4 | In furrow/ banded |
|  |  |  | 5 | Boom on Tractor |
|  |  |  | 6 | Backpack |
|  |  |  | 7 | Hand Spray |
|  |  |  | 8 | Air Blast |
|  |  |  | 9 | Mist Blower /Fogger |
| f | | Have you ever repaired the instrument which are used for the applying pesticides? | 1 | Yes |
|  |  |  | 0 | No |
| g | | Had you used the PPE 1 year before the diagnosis of disease? | 1 | Yes |
|  |  |  | 0 | No |
| h | | Types of PPE used during spray 1 year before the diagnosis of study | 0 | Never |
|  |  |  | 1 | face mask |
|  |  |  | 2 | Gloves |
|  |  |  | 3 | leather gloves |
|  |  |  | 4 | other PPE, Like boot |
|  |  |  | 5 | gas mask |
|  |  |  | 6 | Disposable outer clothes |
|  |  |  | 7 | Chemical resistant rubber gloves |

| 29 | Food Pattern | | | | | |
| --- | --- | --- | --- | --- | --- | --- |
|  | Food items /frequency | UNIT/FREQUENCY | | | | |
|  |  | Never | Times/ day | Times/week | Times/Month | Times/Year |
|  | Rice |  |  |  |  |  |
|  | Brown Rice |  |  |  |  |  |
|  | Wheat |  |  |  |  |  |
|  | Choumin |  |  |  |  |  |
|  | Pasta macaroni spaghetti |  |  |  |  |  |
|  | Sooji |  |  |  |  |  |
|  | Aata roti |  |  |  |  |  |
|  | Bhuteko maize |  |  |  |  |  |
|  | White Bread |  |  |  |  |  |
|  | Brown bread |  |  |  |  |  |
|  | Kodo fapar bajra |  |  |  |  |  |
|  | whole pulse |  |  |  |  |  |
|  | Washed Legumes |  |  |  |  |  |
|  | Sprout |  |  |  |  |  |
|  | Cheakpeas dry peas beans |  |  |  |  |  |
|  | Soyabean |  |  |  |  |  |
|  | Broccoli Cauliflower |  |  |  |  |  |
|  | Cabbage |  |  |  |  |  |
|  | Pumpkin |  |  |  |  |  |
|  | Potato |  |  |  |  |  |
|  | Radish |  |  |  |  |  |
|  | Greenveg |  |  |  |  |  |
|  | Parbal |  |  |  |  |  |
|  | Green Beans Peas |  |  |  |  |  |
|  | Karela |  |  |  |  |  |
|  | Egg plant |  |  |  |  |  |
|  | Tomato |  |  |  |  |  |
|  | Lauka |  |  |  |  |  |
|  | Schoos |  |  |  |  |  |
|  | Lady's finger |  |  |  |  |  |
|  | Ghiraula |  |  |  |  |  |
|  | Rukhkatahar |  |  |  |  |  |
|  | Yam |  |  |  |  |  |
|  | Sweet Potato |  |  |  |  |  |
|  | Taamaa |  |  |  |  |  |
|  | Gundruk |  |  |  |  |  |
|  | Mushroom |  |  |  |  |  |
|  | Carrot |  |  |  |  |  |
|  | Cucumber |  |  |  |  |  |
|  | Orange |  |  |  |  |  |
|  | Grape_fruti |  |  |  |  |  |
|  | Pine apple |  |  |  |  |  |
|  | Guava |  |  |  |  |  |
|  | Jujube_aaru |  |  |  |  |  |
|  | Apple_ pear |  |  |  |  |  |
|  | Alubhakada |  |  |  |  |  |
|  | Lychee |  |  |  |  |  |
|  | Grapes |  |  |  |  |  |
|  | Lemon |  |  |  |  |  |
|  | Banana |  |  |  |  |  |
|  | Mango |  |  |  |  |  |
|  | Pomegranate |  |  |  |  |  |
|  | Watermelon |  |  |  |  |  |
|  | Papaya |  |  |  |  |  |
|  | Mutton |  |  |  |  |  |
|  | Chicken_with_ skin |  |  |  |  |  |
|  | Chicken_without_ skin |  |  |  |  |  |
|  | Chicken mo_mo |  |  |  |  |  |
|  | Sausage |  |  |  |  |  |
|  | Buff |  |  |  |  |  |
|  | Buff mo_mo |  |  |  |  |  |
|  | Buff sausage |  |  |  |  |  |
|  | Pork |  |  |  |  |  |
|  | Pork mo_mo |  |  |  |  |  |
|  | Fried fish |  |  |  |  |  |
|  | Nonfried fish |  |  |  |  |  |
|  | Egg |  |  |  |  |  |
|  | Whole milk |  |  |  |  |  |
|  | Low fat milk |  |  |  |  |  |
|  | Yogurt |  |  |  |  |  |
|  | Milk tea |  |  |  |  |  |
|  | Black tea |  |  |  |  |  |
|  | Milk coffee |  |  |  |  |  |
|  | Black coffee |  |  |  |  |  |
|  | Coke_ pepsi_mountain_dew |  |  |  |  |  |
|  | Fanta sprite |  |  |  |  |  |
|  | Fruit_ juice |  |  |  |  |  |
|  | Canned juice |  |  |  |  |  |
|  | Paneer |  |  |  |  |  |
|  | Pizza |  |  |  |  |  |
|  | Cheese |  |  |  |  |  |
|  | Biscuit |  |  |  |  |  |
|  | Noodles |  |  |  |  |  |
|  | Canned food |  |  |  |  |  |
|  | Peanuts |  |  |  |  |  |
|  | Cashew |  |  |  |  |  |
|  | Almonds |  |  |  |  |  |
|  | walnut |  |  |  |  |  |
|  | Pistachio |  |  |  |  |  |
|  | dried Fruit |  |  |  |  |  |
|  | Bhujia |  |  |  |  |  |
|  | Vegburger |  |  |  |  |  |
|  | Chickenburger |  |  |  |  |  |
|  | Potato Chips |  |  |  |  |  |
|  | Donought |  |  |  |  |  |
|  | Ice cream |  |  |  |  |  |
|  | Chocolate |  |  |  |  |  |
|  | sweets |  |  |  |  |  |
|  | Malpa |  |  |  |  |  |
|  | Swaari |  |  |  |  |  |
|  | Pakauda |  |  |  |  |  |
|  | French Fries |  |  |  |  |  |
|  | Oil |  |  |  |  |  |
|  | Butter |  |  |  |  |  |
|  | ghee |  |  |  |  |  |
|  | sugar |  |  |  |  |  |
|  | Jaggary |  |  |  |  |  |
|  | jam |  |  |  |  |  |
|  | Pickels |  |  |  |  |  |
